#  Evaluating Autonomic Parameters: The Role of Sleep ‎Duration in Emotional Responses to Music ‎

**Published:** 2016-01

**Authors:** Atefeh Goshvarpour‎, Ataollah Abbasi, Ateke Goshvarpour‎

**Affiliations:** **1.** Computational Neuroscience Laboratory, Department of Biomedical Engineering, Faculty ‎of Electrical Engineering, Sahand University of Technology, Tabriz, Iran

**Keywords:** Emotion‎, Physiological Signals, Power Spectral Density, Signal Processing, Sleep

## Abstract

**Objective: **It has been recognized that sleep has an important effect on emotion processing. The aim ‎of this study was to investigate the effect of previous night sleep duration on autonomic ‎responses to musical stimuli in different emotional contexts.‎

**Method:** A frequency based measure of GSR, PR and ECG signals were examined in 35 healthy ‎students in three groups of oversleeping, lack of sleep and normal sleep. ‎

**Results:** The results of this study revealed that regardless of the emotional context of the musical ‎stimuli (happy, relax, fear, and sadness), there was an increase in the maximum power of ‎GSR, ECG and PR during the music time compared to the rest time in all the three ‎groups. In addition, the higher value of these measures was achieved while the ‎participants listened to relaxing music. Statistical analysis of the extracted features ‎between each pair of emotional states revealed that the most significant differences ‎were attained for ECG signals. These differences were more obvious in the participants ‎with normal sleeping (p<10-18). The higher value of the indices has been shown, ‎comparing long sleep duration with the normal one.‎

**Conclusion**: There was a strong relation between emotion and sleep duration, and this association can ‎be observed by means of the ECG signals.‎

Sleep is crucial for the body and its functions. Different Sleep durations (short or long) ‎have profound unwanted side effects on many characteristics of physiological function, ‎mood, cognition, alertness and memory. Some consequences of insufficient sleep are as ‎follows: 

1.    sleepiness, 

2.    tiredness, 

3.    negative effects on attention, mood, and behavior,

4.    And emotional and behavioral complications (1). 

For example, oversleeping, which is ‎defined as sleeping more than the body needs, may cause fatigue and lethargy, and ‎makes it difficult to sleep well at night. Furthermore, some negative effects of ‎oversleeping on subjective mood, performance, sleepiness and vigilance have been ‎reported (2-3).

There is no scientific consensus on the sufficient sleep duration. Some researcher’s ‎claimed that eight hours of sleep is required for young adults, but others believe ‎otherwise. They believe that some individuals may need only five or six hours of sleep ‎per night. Bonnet and Arand (4) concluded that the best way to regulate an individual’s ‎need for sleep is to get some sleep when exhausted and sleepy, and to get up without ‎any alarm when feeling reinvigorated. Although many studies applied some thresholds ‎for sleep duration, these values may vary based on the physiological outcome of interest ‎‎(5). On the other hand, it has been shown that different participants may need variable ‎sleep amount to obtain optimal performance in their daily activities (6). Therefore, in the ‎current protocol, the ratio of the previous nights’ sleep to the routine one was calculated ‎and applied as an index of sleep categories for all the participants. ‎

Some clinical evidence proposed that emotion and sleep interact with each other (7). It ‎is also increasingly recognized that sleep has an important effect on emotion processing ‎‎(8). The important impact of sleep duration on emotional functioning and cognitive ‎performance in children has also been reported (9). Sleep-deprivation yields decrements ‎in emotion reactivity, as well as reduction in the sensitivity to positive stimuli and ‎amplification in the sensitivity to negative stimuli (10-12). To regulate the emotions, ‎neuropsychological evidences recommended that both sleep quantity and its’ quality are ‎vital for the best brain functioning (13). Recently, it has been suggested (10) getting ‎enough sleep is important for optimal processing and evaluation of emotion, and ‎participants with insufficient sleep may be biased when processing negative valence ‎stimuli.‎

It was found that short and long sleep durations influence the autonomic nervous ‎system (ANS) and human vital signs (14-15). For instance, a strong association was ‎found between sleep duration and mental health condition by means of ‎electrocardiographic (ECG) outcomes (16).‎

Although ANS activity during emotional stimuli is one of the major matters that ‎researchers pay much attention to (17), to date no attempt has been made to investigate ‎the relation between emotions and sleep duration by means of autonomic measures.‎

In this study, the effects of sleep duration on autonomic emotional responses were ‎addressed, by means of Galvanic skin responses (GSR), electrocardiogram (ECG) and ‎pulse rate (PR) recordings.‎

## Materials and Method

***Data Selection:***

To understand the physiological changes elicited by music in different sleep patterns ‎some measures of emotional responses were examined in three groups of students with a ‎single night of oversleeping, lack of sleep and normal sleep through physiological ‎signals. Three groups of students were selected via empirical thresholds on the ratio of ‎their sleep as follows (1): 


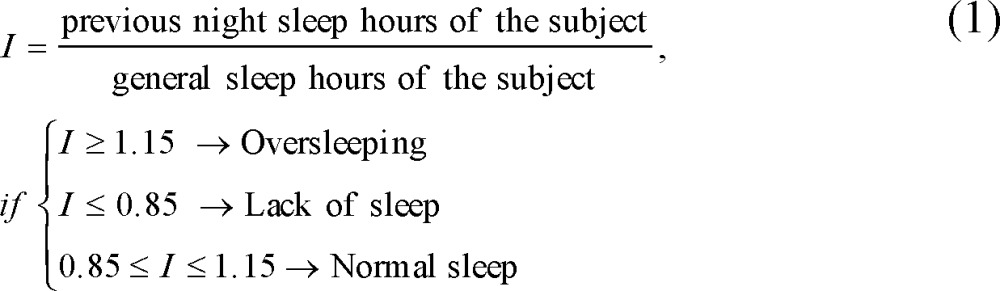


ECG, GSR and PR signals of 35 college students attending Sahand University of ‎Technology, who were categorized into three groups of oversleeping (13 participants ‎including 5 female and 8 male; age range of 22.38±1.5), lack of sleep (9 participants ‎including 4 female and 5 male; age range of 21.67±1) and normal sleep (13 participants ‎including 5 female and 8 male; age range of 22.69±1.44) were collected. All the ‎participants were Iranian students; and were asked to read and sign a consent form, ‎which they only signed if they agreed to take part in the study. ‎

The experimental design consisted of two different stages: First, the initial baseline ‎measurement was carried out for two minutes with eyes closed. Then, the participants ‎listened to the blocks of music. They were instructed to lie in supine position and try to ‎remain motionless. Physiological signals were recorded for about 15 minutes while the ‎participants listened to the music. All tests were performed in a controlled temperature ‎and light. The mean temperature of the room was about 23oC. Musical pieces were ‎presented via KMPlayer software, with headphone at a comfortable volume. . ‎

The ECG signals (Lead I), GSR and PR of all participants were recorded in ‎Computational Neuroscience Laboratory of Sahand University of Technology using 16-‎channel Power Lab (manufactured by AD Instruments). A digital notch filter was ‎applied to the data at 50 Hz to remove any artifacts caused by alternating current line ‎noise. The sampling rate was 400 Hz.‎

Fifty-six short musical excerpts, which were validated by Vieillard et al., were selected ‎as stimuli (18). These excerpts were used to express happiness, sadness, peacefulness ‎and fear; there were fourteen stimuli per category, and they varied on arousal ‎‎(relaxing/stimulating) and valence (pleasant/unpleasant) dimensions. They consisted of a ‎melody with accompaniment composed in piano timbre, and followed the rules of the ‎Western tonal system (18).‎

‎***Frequency Analysis***

**Figure 1:**
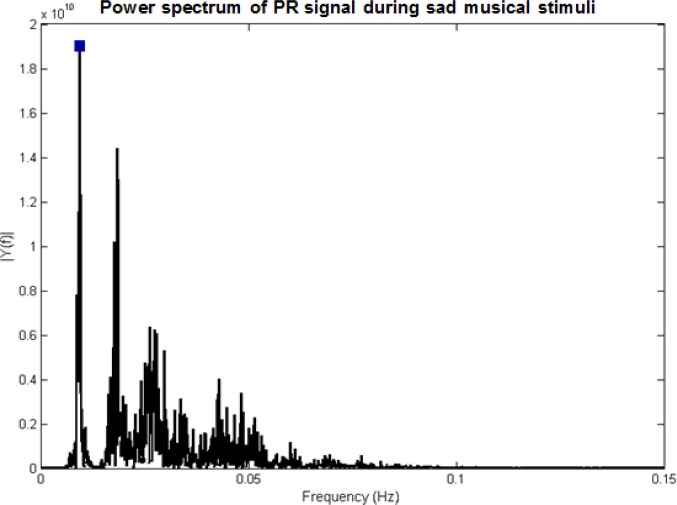
Power Spectrum of PR Signal during Sad Musical Stimuli. The maximum power of PR was indicated by a blue rectangle (subject 15).

Applying the Fast Fourier transform (FFT) (19), power spectral density (PSD) of each ‎signal was estimated. Then, the maximum value of the power was considered as an ‎indicator of emotional reaction, which is schematically demonstrated in Figure 1.‎

## Results

**Figure 2:**
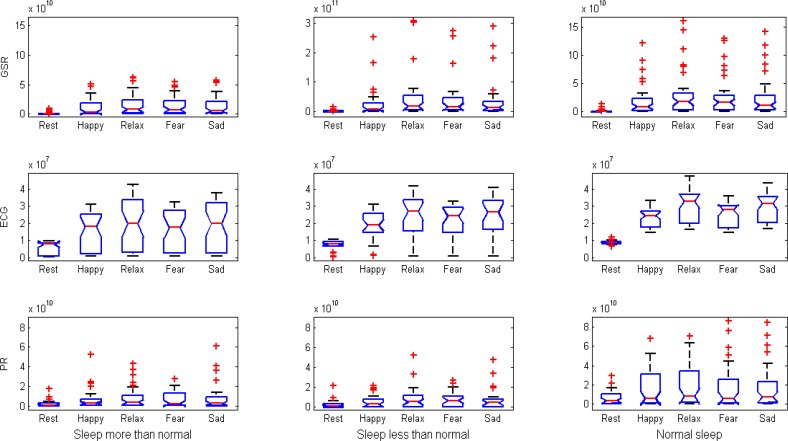
The Variation of the Maximum Power of GSR (first row), ECG (second row) and PR (third row) during the rest time and during the music with four different categories of emotions including happy, relax, fear and sadness. First column: Long sleep duration; second column: Short sleep duration; third column: Normal sleep duration

This experiment was planned to test the effects of different sleep durations on the emotional responses ‎of college students. GSR, ECG and PR of 35 healthy college students while listening to music clips with ‎different emotional contexts, were examined in three groups: Oversleeping, lack of sleep, and normal ‎sleep. Figure 2 demonstrates the maximum power of signals during the music (happy, relax, sad and fear) ‎and the rest time.‎

These results confirmed that all pairs of emotions could be accurately separated by applying ECG signals. In contrast, there were no significant differences between each pair of emotional states of the participants with normal sleeping and oversleeping by means of PR signals. In addition, no significant changes were detected between sadness and fear, using GSR and PR parameter. By applying ECG signal, we found that the most significant changes occurred when participants had normal sleep duration; however, these values were lower in participants with short sleep duration.

**Table 1:** Wilcoxon Mann- Whitney Values between the Maximum Power of Normal vs. Oversleeping and Normal vs. Less Sleep for GSR, ECG and PR

	**Normal vs. oversleeping**					**Normal vs. lack of sleep**				
	**Rest**	**Happy**	**Relax**	**Fear**	**Sad**	**Rest**	**Happy**	**Relax**	**Fear**	**Sad**
**GSR**	0.59	0.12	0.12	0.11	0.13	0.88	0.82	0.95	0.71	0.88
**ECG**	5.4×10^-5 *^	0.0041^*^	0.0008^*^	0.001^*^	0.0024^*^	0.041^*^	0.043^*^	0.039^*^	0.05^*^	0.049^*^
**PR**	0.02	0.05^*^	0.05^*^	0.06	0.03^*^	0.023^*^	0.045^*^	0.13	0.11	0.064

GSR: Galvanic Skin Response, ECG: Electrocardiogram, and PR: Pulse Rate.

**Table 2:** The Statistical Analysis (t-test) of the Maximum Power between Each Pair of Emotional States

	**Relax vs. Fear**	**Relax vs. Happy**	**Relax vs. Sad**	**Happy vs. Fear**	**Happy vs. Sad**	**Sad vs. Fear**
**Normal Sleep**						
GSR	0.01^*^	0.001^*^	0.004^*^	0.003^*^	0.001^*^	0.19
ECG	1.15×10^-12 *^	5.79×10^-10 *^	0.0001^*^	6.32×10^-06 *^	1.46×10^-09 *^	1.93×10^-11 *^
PR	0.44	0.13	0.21	0.57	0.8462	0.69
**Oversleeping**						
GSR	0.01^*^	0.0005^*^	0.004^*^	0.006^*^	0.002^*^	0.17
ECG	5.18×10^-8 *^	1.36×10^-6 *^	0.007^*^	0.0007^*^	6.34×10^-7 *^	1.32×10^-9 *^
PR	0.09	0.19	0.87	0.60	0.21	0.47
**Sleep Less than Normal**						
GSR	0.013^*^	0.04^*^	0.05^*^	0.08	0.04^*^	0.56
ECG	3.67×10^-7 *^	8.89×10^-6 *^	0.002^*^	0.001^*^	5.11×10^-6 *^	5.60×10^-8 *^
PR	0.38	0.02^*^	0.40	0.005^*^	0.037^*^	0.79

## Discussion

There is an astonishing lack of studies investigating the effects of sleep duration on emotional responses. ‎The current study utilized an experimental design to test the effects of the previous night’s sleep duration ‎on young college students’ emotion responses as assessed objectively applying GSR, ECG and PR. To ‎our knowledge, this was the first experimental study to examine the effects of different sleep ratios on ‎the emotional response of young participants, using physiological measurement. The results ‎demonstrated that the lower amount of the mean and standard deviation of the maximum power of ‎GSR, ECG, and PR were found during the rest time (p<0.01). However, the heightened values were ‎presented during the relaxing music stimuli. The higher value of the indices was seen in participants with ‎normal sleeping compared to those with long sleep duration. Applying ECG signals, all pairs of emotions ‎could be accurately separated from each other, but these differences were not significant for PR signals. ‎Moreover, no significant changes were observed between sad and fear music categories in GSR and PR. ‎Therefore, it can be concluded that among the mentioned signals, ECG was the best for the study of ‎sleep effects on the emotional responses. ‎

Previously, an association was reported between modestly increased risk of coronary events and short ‎and long self-reported sleep durations (20). In addition, assessing autonomic functions and sleep ‎deprivation, some similar results have been obtained by earlier studies. Moreover, a relationship was ‎found between high blood pressure and sleep deprivation and lack of sleep (14, 21), which emphasizes ‎the impact of sleep disturbances on cardiovascular regulation. ‎

A few attempts have been made to determine the effects of sleep‏ ‏constraints on overall emotion. ‎Participants with sleep deprivation stated that not only their crying reactions had increased during the sad ‎scenes of the video clips, but also their irritability and impatience increased and their acceptance for ‎frustration decreased during performing tedious computer tasks (1). Generally, it has been found that ‎following short sleep duration, there is a decrease in conscious control or inhibition over emotions. Berger ‎et al. (7) studied the links between sleep and emotion processing in 30- to 36-month-old children by ‎applying emotion eliciting pictures (5 positive, 3 negative, and 3 neutral) and completed puzzles (1 ‎solvable, and 1 unsolvable). They found that when sleep is restricted, less mistakes in response to neutral ‎pictorial stimuli, more negativity to negative and neutral pictures, and less positivity to positive pictures ‎occurred. These findings suggested that sleep has a main influence on young children’s response to their ‎world (7). One of the shortcomings of this study was evaluating a valence aspect of emotions, separately. ‎To overcome this limitation, in the current study, both dimensions of valence and arousal were examined ‎simultaneously. Consequently, four different emotional categories (Happy, relax, fear, and sadness) were ‎considered.‎

## Limitations

Some limitations of this investigation should be noted. The limited size of the sample may reduce the ‎generalizability of the findings to other populations. In addition, there was a lack of information about ‎the long-term effects of oversleeping or insufficient sleep on the emotional responses of college students, ‎since the restricted accessible data have addressed only the short-term effects of short and long sleep ‎duration. Therefore, the long term effects of different sleep patterns on emotions in young adults should ‎be studied in future researches.‎

## Conclusion

In summary, the data suggest a strong relation between emotion and sleep duration and this association ‎can be observed by means of the ECG parameters.‎
